# Rapamycin-induced protein dimerization as a tool for *C. elegans* research​

**DOI:** 10.17912/W2BH3H

**Published:** 2018-03-20

**Authors:** Sriyash Mangal, Jeffrey Zielich, Eric J. Lambie, Esther Zanin

**Affiliations:** 1 Department of Cell and Developmental Biology, Ludwig-Maximillians-University, Munich, Planegg-Martinsried, Germany.

**Figure 1. After injection of rapamycin, cytosolic mCherry::FKBP12 dimerizes with FRB::GFP::PH and translocates to the plasma membrane.**
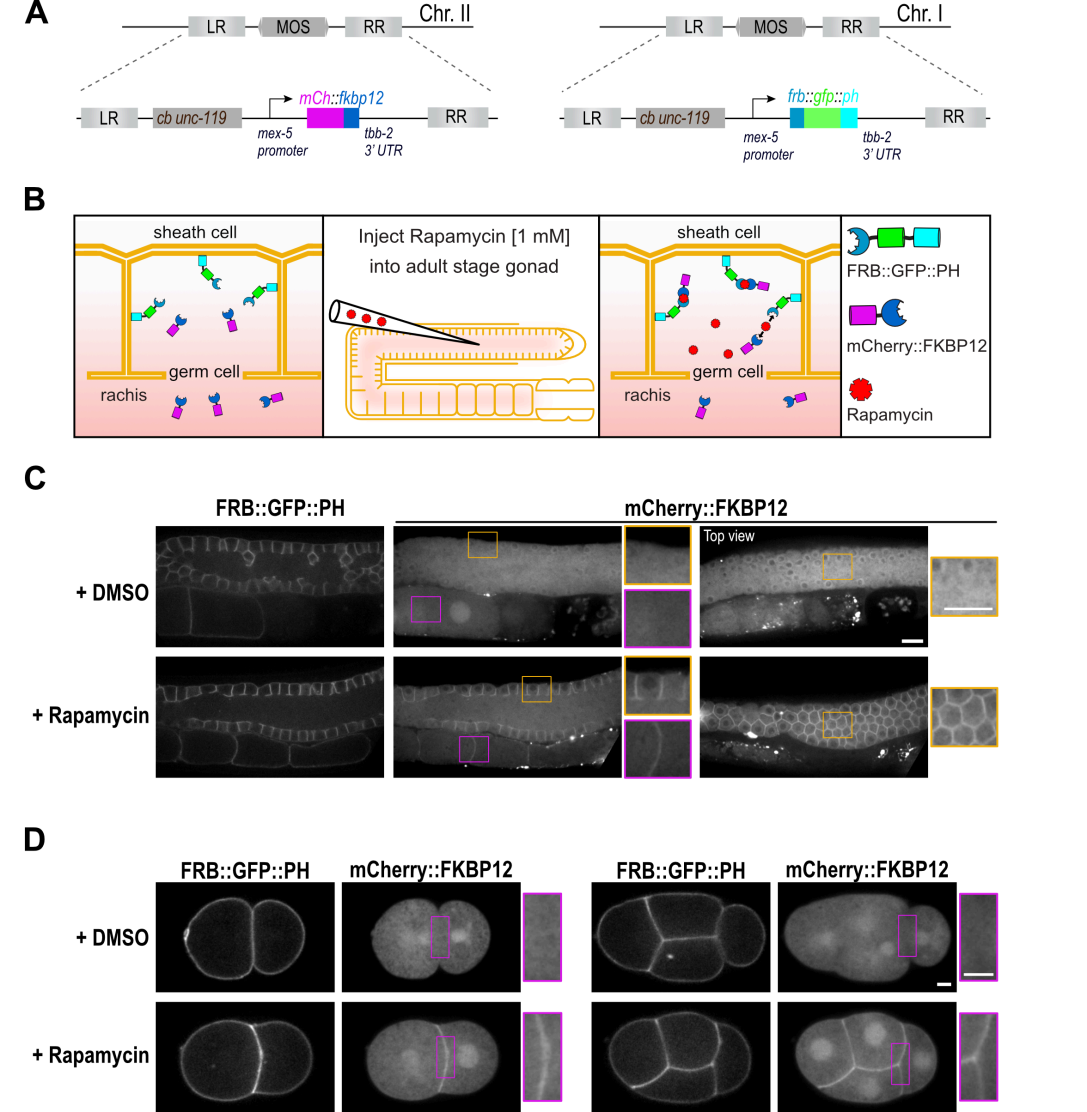
**(A)** The mCherry-tagged FKBP12 and GFP-PH-tagged FRB transgenes under control of the *mex-5* promoter and *tbb-2* 3′ UTR were integrated into chromosome II and chromosome I, respectively. **(B)** Schematic representation of rapamycin-induced heterodimerization of FRB and FKBP12 fusion proteins in the *C. elegans* gonad. The GFP-tagged FRB domain is tethered to the plasma membrane by the PH domain and the mCherry-tagged FKBP12 is present in the cytoplasm. After injection of 1 mM rapamycin into the gonad, heterodimers of FRB and FKBP12 fusion proteins form and mCherry::FKBP12 translocates to the plasma membrane. **(C)** Confocal images of germ line of an adult worm expressing FRB::GFP::PH and mCherry::FKBP12 2-3 hours after injection with DMSO (*n* = 19) or 1 mM rapamycin (*n* = 29) into the gonad. *n* = number of gonads. Yellow insets highlight the plasma membrane of gonadal germ cells and magenta insets highlight the plasma membrane of oocytes. Scale bar 10 μm. **(D)** Confocal images of two-cell and four-cell embryos imaged approximately 2-3 hours after the gonads were injected with DMSO (2-cell embryo *n* = 10; 4-cell embryo *n =* 9) or 1 mM rapamycin (2-cell embryo *n* = 8; 4-cell embryo *n =* 9). Insets highlight the plasma membrane between the blastomeres. Scale bar 5μm.

## Description

**​**Induced protein dimerization is an invaluable tool in cellular biology to study protein function. Induced dimerization has been widely used to modulate enzymatic activity as well as expression and localization of proteins (Voß *et al.*, 2015; DeRose *et al.*, 2013). A popular method employs the chemical dimerizer rapamycin to induce binding between the FRB domain of the mTOR kinase and the FKBP12 protein (FK506 binding protein 12 kDa) (Putyrski and Schultz, 2012). Rapamycin-induced protein dimerization has been extensively used in cell culture systems and yeast to control RhoA GTPases signaling (Inoue *et al.*, 2005), protein stability (Janse *et al.*, 2004) or phosphoinosite composition of the plasma membrane (Ueno *et al.*, 2011). The small nematode *C. elegans* is a popular model system for cell biology, however to our knowledge, rapamycin-induced protein dimerization has not been successfully used in this organism. To establish the rapamycin-induced protein dimerization technique in *C. elegans* we codon-optimized the human FRB and FKBP12 domains and introduced one intron to ensure high expression of the transgenes. The FRB domain was fused to GFP and the plekstrin homology domain (PH) (Audhya *et al.*, 2005) at the C-terminus to anchor it at the plasma membrane and the FKBP12 domain was fused to mCherry (Figure 1A, B). Both transgenes are controlled by the *mex-5* promoter and *tbb-2* 3’UTR to ensure high and ubiquitous expression of the fusion proteins. After we generated single-copy integrations using MosSCI (Frøkjær-Jensen *et al.*, 2008) both strains were crossed together. As expected FRB::GFP::PH localizes to the plasma membrane in the germ line and early embryos and mCherry::FKBP12 is present in the cytoplasm and the nucleus (Figure 1C, D). To induce binding of the FRB and FKBP12 domains and thereby translocation of the mCherry::FKBP12 to the plasma membrane, we injected 1 mM rapamycin into the pachytene region of the germ line. Upon rapamycin injection, mCherry::FKBP12 translocated from the cytoplasm to the plasma membrane in all germ lines (Figure 1C) and early embryos analyzed (Figure 1D). In control worms injected with DMSO, translocation of mCherry::FKBP12 to the plasma membrane was not observed (Figure 1C, D). As expected in rapamycin-injected worms expressing only the mCherry::FKBP12 transgene, no translocation of mCherry::FKBP12 to the plasma membrane was visible (*n* = 7 gonads). Importantly after rapamycin injection we did not observe alterations in gonad morphology (*n* = 29 gonads) and early embryonic divisions (*n* = 17 embryos) or embryonic lethality (+DMSO 281/0; +rapamycin 302/0; number of viable/dead progeny) validating applicability of our system for future cell biological studies. mCherry::FKBP12 localizes to the nucleus and the cytoplasm and therefore dimerization can be induced in both compartments. In case dimerization will be used to selectively target proteins to the nucleus or cytoplasm additional modifications of the presented system will be required. In summary, we establish a rapamycin-inducible dimerization system in *C. elegans* and demonstrate that it can be used to target a protein of interest to a specific subcellular region in the germ line and in early embryos. Our system can be directly used to target any protein to the plasma membrane of germ cells or early embryos by fusing it to the FKBP12 domain. Moreover, it can be easily modified to target proteins to different subcellular locations and to control protein activity or stability.

**Table 1:** Used *C. elegans* strains

**Table:** 

**Strain Name**	**Genotype**	**Reference**
EG6699	*ttTi5605* II; *unc-119(ed3)* III; oxEx1578	Frøkjær-Jensen *et al.*, 2008
EG8078	*oxTi185* I; *unc-119(ed3)* III	Frøkjær-Jensen *et al.*, 2014
ZAN87	*estSi50[pEZ156;pmex-5::frb::gfp::ph::tbb2; cb-unc-119(+)]*I; *unc-119(ed3)* III	This study.
ZAN98	*estSi54[pEZ159;pmex-5::mCherry::fkbp12::tbb2; cb-unc-119(+)]*II; *unc-119(ed3)* III	This study.
ZAN101	*estSi50[pEZ156;pmex-5::frb::gfp::ph::tbb2; cb-unc-119(+)]*I; *estSi54[pEZ159;pmex-5::mCherry::fkbp12::tbb2; cb-unc-119(+)]*II; *unc-119(ed3)* III	This study.

## Reagents

**Generation of *C. elegans*** **strains** *C. elegans* strains were grown at 20oC on NGM agar plates according to standard procedures (Stiernagle, 2006). Gibson cloning (E2611; NEB) was used to construct transgenes encoding FRB::GFP::PH and mCherry::FKBP12 in pCFJ350. cDNA sequences of human FRB (NM_004958) and FKBP12 (CR542168) were codon-optimized (Redemann *et al.*, 2011) for expression in *C. elegans* and introns were introduced between amino acids 25(K)-26(G) and amino acids 35(K)-36(K), respectively and DNA was synthesized by Eurofins Genomics. In the FRB domain ‘threonine’ 2098 was mutated to ‘leucine’ which allows the binding to rapamycin derivatives that do not interact with mTOR kinase (Bayle *et al.*, 2006). Single-copy insertions of FRB::GFP::PH and mCherry::FKBP12 were generated on chromosomes I and II, respectively, using the MosSCI method (Frøkjær-Jensen *et al.*, 2008; 2014). Expression of the transgenes was controlled by the *mex-5* promoter and the *tbb-2* 3′ UTR (Zeiser *et al.*, 2011). Finally, the two *C. elegans* strains *frb::gfp::ph* and *mCherry::fkbp12* were crossed together to obtain expression of both transgenes in one strain.

**Rapamycin injection**10 mM stock of rapamycin (Cayman Chemical, 13346) was prepared in DMSO and stored at -20oC. The two gonad arms of adult worms were injected with 1 mM rapamycin or 10% DMSO (control) diluted to their final concentration in water.

**Fluorescence Microscopy**For imaging *C. elegans* embryos, adult worms were dissected 1.5 to 2 hours after injection in a 4-μl drop of M9 buffer on an 18 × 18-mm coverslip, and the coverslip was inverted onto a 2% agarose pad. For imaging adult *C. elegans* worms, a few animals were mounted on a 10% agarose pad (prepared in 0.6x M9 buffer) with 1 ul of immobilizing 0.10 micron beads (00876, Polysciences) (Kim *et al.*, 2013). An 18 × 18-mm coverslip was placed on top and the surrounding region of the agarose pad was filled with mineral oil to prevent shrinking of the agarose pad. All images were acquired at 25°C on an eclipse Ti spinning disk confocal (Nikon), which was controlled by NIS Elements 4.51 and equipped with a 100x 1.45-NA Plan-Apo-chromat oil immersion objective, a 488-nm and 561-nm laser line, and an Andor DU-888 X-11056 camera.
