# Erratum: Rapamycin-induced protein dimerization as a tool for C. elegans research​

**DOI:** 10.17912/micropub.biology.000194

**Published:** 2019-12-02

## Description

Mangal, S; Zeilich, J; Lambie, EJ; Zanin, E (2018). Rapamycin-induced protein dimerization as a tool for *C. elegans* research​. microPublication Biology. 10.17912/W2BH3H published online March 20, 2018; corrected after publication Dec 2, 2019.

In the version of the microPublication originally published online, the last name of the second author was misspelled. The correct spelling is Zielich. This mistake has been corrected online. The spelling was correct in the original PDF version.

